# Factors related to baseline CD4 cell counts in HIV/AIDS patients: comparison of poisson, generalized poisson and negative binomial regression models

**DOI:** 10.1186/s13104-021-05523-w

**Published:** 2021-03-25

**Authors:** Maryam Farhadian, Younes Mohammadi, Mohammad Mirzaei, Nasrin Shirmohammadi-Khorram

**Affiliations:** 1 Department of Biostatistics, School of Public Health, Hamadan University of Medical Sciences, Hamadan, Iran; 2 Research Center for Health Sciences, Hamadan University of Medical Sciences, Hamadan, Iran; 3 Department of Epidemiology, School of Public Health, Hamadan University of Medical Sciences, Hamadan, Iran; 4 Modeling of Noncommunicable Diseases Research Center, Hamadan University of Medical Sciences, Hamadan, Iran; 5 Hamadan Health Center, Hamadan University of Medical Sciences, Hamadan, Iran

**Keywords:** CD4 Lymphocyte Count, HIV, AIDS, Disease Progression, Statistical Model

## Abstract

**Objective:**

CD4 Lymphocyte Count (CD4) is a major predictor of HIV progression to AIDS. Exploring the factors affecting CD4 levels may assist healthcare staff and patients in management and monitoring of health cares. This retrospective cohort study aimed to explore factors associated with CD4 cell counts at the time of diagnosis in HIV patients using Poisson, Generalized Poisson, and Negative Binomial regression models.

**Results:**

Out of 4402 HIV patients diagnosis in Iran from 1987 to 2016, 3030 (68.8%) were males, and the mean age was 34.8 ± 10.4 years. The results indicate that the Negative Binomial model outperformed the other models in terms of AIC, log-likelihood and RMSE criteria. In this model, factors include sex, age, clinical stage and Tuberculosis (TB) co-infection were significantly associated with CD4 count (P < 0.05).

**Conclusion:**

Given the effect of age, sex, clinical stage and stage of HIV on CD4 count of the patients, adopting policies and strategies to increase awareness and encourage people to seek early HIV testing and care is advantageous.

## Introduction

AIDS (Acquired Immunodeficiency Syndrome) disease is a threating factor for human life in the world [1–5]. In the beginning of the HIV (Human Immune System Virus) epidemic, about 75 million people infected with the virus worldwide [6, 7]. Despite World Health Organization (WHO) has predicted the new cases of HIV and death from AIDS in 2020 will be reduced to 500,000 [8], it will be remained as one of the massive challenges which affects all levels of social, family, and individual activities of mankind [1, 9].

Decelerating the progression of HIV to AIDS is a crucial measure to deal with the disease. Accordingly, number of CD4 lymphocytes (CD4) in HIV-infected individuals is a principal indicator of HIV progression and death from AIDS [10–12] and that lower CD4 cell count level indicates that the immune system may be compromised [10]. WHO emphasizes on the important role of CD4 counting in assessing the initial status of the disease as well as making appropriate decisions and care for patients with advanced HIV. Therefore, in care systems where access to Antiretroviral Therapy (ART) may be limited, patients with CD4 cell counts less than 350 cells per cubic millimeter are prioritized [11]. There is a correlation between CD4 cell count and risk of death [13], life expectancy [14] and adherence to treatment [15]. The decreased CD4 cells increases the risk of other infectious diseases and clinical symptoms associated with HIV [16]. Specifically, CD4 cell counts less than 200 cells per cubic millimeter is used for description of advanced HIV disease and as the critical threshold for the risk of death [8, 13, 17, 18].

The identification of factors affecting CD4 cell counts at the early stage of treatment for HIV patients may be plays an important role in their care program and their survival [19]. In addition to HIV, other factors can affect the number of CD4 cells. The influence of factors such as infection with other viruses, ethnicity, geographic location, genetics, route of transmission, nutrition, pregnancy, stress, smoking status and drug use on CD4 cell counts have been investigated [1, 10, 20–24]. Therefore, modeling and analysis of factors affecting the number of CD4 cells in AIDS research is important. Several generalized linear models are available for modeling the counting data including Poisson and Negative Binomial (NB) as well as Generalized Poisson regression models (GPR) [12, 19, 24–26]. In recent years, these models have been used extensively in epidemiology and health studies [12, 19, 26–28].

This study aimed to compare the performance of Poisson, Negative Binomial and GPR models to evaluate the effect of different demographic factors on CD4 count at the time of diagnosis in HIV patients.

## Main text

### Data source

Data from this registry-based retrospective cohort study included information on all newly diagnosed HIV / AIDS patients in 158 Behavioral Disease Counseling Centers in 31 provinces of Iran from 1987 to 2016 [29, 30]. Inclusion criteria are as follows: HIV positive, having CD4 cell count at the time of diagnosis (measured up to 3 months after diagnosis), and not receiving ART treatment before determining CD4 cell count.

The initial CD4 cell count was considered as a response variable. The potential predictor variables for this study were age group (< 30,30–40,40–50, > 50), gender (male, female), educational status (illiterate/primary school, secondary school, high school, academic, unknown), material status (single, married, widow, divorce, unknown), job (employed, unemployed, unknown), transmission way (injecting drug users, unsafe sexual, mother to child, blood transfusion and unknown), year of diagnostic (before 2006, 2006–2011, after 2011), TB co-infection (yes, no), WHO clinical stage disease (stage I, stage II, stage III, stage IV, unknown).

The following two scenarios were used to evaluate the impact of missing observations on independent variables: firstly to handle the missing data assuming missing at random (MAR) pattern, we applied MI using five imputed data sets. Secondary missing observations in each independent variable were considered in a separate category with unknown name (as reported in the Tables 1 and 2). In estimating factors associated with baseline CD4 count, results were similar in both scenarios. Therefore results of study were reported based on second scenario.

**Table 1:** General characters of the participated based on the CD4 count

Variable	N (%)	Mean	SD	Min	Max
All	4402 (100)	344.77	280.57	9.00	1492.00
Age (year)					
< 30	1445 (32.8)	449.08	311.78	9.00	1492.00
30–40	1856 (42.2)	308.03	246.55	9.00	1468.00
40–50	784 (17.8)	281.10	260.01	9.00	1445.00
> 50	317 (7.2)	241.88	222.42	9.00	1305.00
Sex					
Female	1372 (31.2)	388.65	292.11	9.00	1475.00
Male	3030 (68.8)	324.90	272.93	9.00	1492.00
Marital status					
Single	1429 (32.5)	368.82	292.58	9.00	1492.00
Married	2133 (48.5)	337.22	266.38	9.00	1445.00
Widow	508 (11.5)	307.27	294.28	9.00	1468.00
Divorce	265 (6)	333.14	275.87	10.00	1305.00
Unknown	67 (1.5)	308.63	336.12	9.00	1330.00
Education					
Illiterate	319 (7.2)	330.55	265.58	9.00	1492.00
Primary School	1020 (23.2)	334.22	272.74	10.00	1468.00
Secondary School	1261 (28.6)	346.34	274.36	9.00	1439.00
High School	919 (20.9)	358.21	286.53	9.00	1476.00
Academic	274 (6.2)	344.16	244.40	10.00	1164.00
Unknown	609 (13.8)	346.64	303.51	9.00	1441.00
Job					
Employed	1521 (34.6)	345.21	285.52	9.00	1492.00
Unemployed	1740 (39.5)	347.36	280.20	9.00	1445.00
Unknown	1141 (25.9)	340.24	274.62	9.00	1468.00
Transmission					
Injecting drug users	122 (2.8)	618.61	424.31	9.00	1492.00
Unsafe sexual	2104 (47.8)	324.54	268.54	9.00	1476.00
Mother to child	1646 (37.4)	370.39	278.25	9.00	1439.00
Blood transfusion and Unknown	530 (12)	370.39	282.44	9.00	1212.00
WHO clinical stage					
Stage 1	1997 (45.4)	420.55	278.69	10.00	1492.00
Stage 2	594 (13.5)	333.39	275.42	9.00	1476.00
Stage 3	575 (13.1)	181.67	185.77	9.00	1200.00
Stage 4	372 (8.5)	157.14	197.81	9.00	1475.00
Unknown	846 (19.6)	366.77	288.37	9.00	1445.00
Year of HIV Diagnosis					
Before 2006	530 (12)	432.85	280.12	18.00	1476.00
2006–2011	1388 (31.5)	325.31	269.89	9.00	1441.00
After 2011	2484 (56.5)	336.85	283.19	9.00	1492.00
TB co-infection					
No	4142(94.1)	350.03	281.17	9.00	1492.00
Yes	260(5.9)	260.91	257.28	9.00	1311.00

**Table 2:** Comparison of three models in terms of Log likelihood and AIC Statistic

Model	Log likelihood	AIC Statistic	RMSE^*^
Generalized Poisson Regression	− 30,508.67	13.86	1.052
Poisson Regression	− 450,409.80	204.64	13.22
Negative Binomial Regression	− 29,948.06	13.54	2.61

RMSE: root mean square error

It is necessary to mention that this data are routinely collected from all patients by a registration system. Therefore, consent was not obtained from patients but study protocol was approved by the Ethics Committee of Hamadan University of Medical Sciences with IR.UMSHA.REC.1398. 406.

## Method

### Statistical models

Poisson distribution is the standard distribution for count data [31]. Let *y*_i_ be a random variable that indicates the baseline CD4 cells counts (cells/mm^3^) of ith individual. Assume that *y*_i_ follows a Poisson distribution with mean *θ*_i_ which related to a set of predictors. Hence, the probability of observing any specific count *y*_i_ is given by the following formula [27].

$$f(y_{i} ;\theta_{i} ) = \frac{{\theta_{i}^{{y_{i} }} e^{ - \theta i} }}{{y_{i} !}}, \quad y_{i} = 0,1,2, \ldots$$

It is assumed that the mean value *θ*_i_ depends on a set of predictor variables, such that $\theta_{i} = \exp ( {\sum\nolimits_{j} {x_{ij} \beta_{j} } } )$ where xi is a covariate vector (age, sex, educational level, …) and β is a vector of unknown regression parameters which should be estimated.

The Poisson distribution assumes that the mean and variance is equal that is referred to as the equidispersion [31]. The phenomenon of large variance relative to the mean is called over-dispersion. This leads to inaccurate estimation of the regression standard errors and the too narrow confidence intervals. In the over-dispersed distribution, a negative binomial regression model can be proposed instead. By assuming *θ*_i_ to be random variable with gamma distribution by mean $E(\theta_{i} ) = \alpha_{i}$ and ${\text{var}} (\theta_{i} ) = r\alpha_{i}$, it can be demonstrated that the marginal distribution of *y*_i_ follows a negative binomial distribution with mean *α*_i_ and variance $\frac{{\alpha_{i} }}{{1 + r\alpha_{i} }}$ where, r denotes the dispersion parameter and $\alpha_{i} = \exp ( {\sum\nolimits_{{}} {x_{ij} \beta_{j} } } )$ .

If r = 0, indicates that no unobserved heterogeneity which leading to Poisson; and if r > 0, then the variance will be larger than mean and over-dispersion has occurred [27, 31].

Another alternative model for data with an over- dispersion event is GPR. Unlike the negative binomial distribution used only in the case of over dispersion, the GPR model can be used for modeling either over-dispersed or over-dispersed counts data sets. The probability density function of the GPR model for the CD4 counts is given by [25, 31]:

$$f(y_{i} ;\theta_{i} ) = \frac{{\alpha_{i} }}{{1 + r\alpha_{i} }}\left( {\frac{{\alpha_{i} \left( {1 + ry_{i} } \right)}}{{1 + ry_{i} }}} \right)^{{y_{i} - 1}} \exp \left( { - \frac{{\alpha_{i} \left( {1 + ry_{i} } \right)}}{{1 + ry_{i} }}} \right)\frac{1}{y!}, \quad y_{i} = 0,1,2,...$$

where the parameter r measures is a dispersion in the data, mean $\alpha_{i} = \exp ( {\sum\nolimits_{j} {} x_{ij} \beta_{j} } )$ and variance $\alpha_{i} (1 + ry_{i} )^{2}$ .

### Models comparison

Akaike information criterion AIC = − 2logL + k where L and k represent the likelihood function and the number of parameters, respectively was used to compare the models [32]. Lower AIC is considered as a better fit of model. Also, the root mean square error of residual (RMSE) was used for model evaluation. The significance level was set to 0.05. All statistical analyses were done using SPSS 23.0 and Stata14 software.

## Results

Descriptive characteristics of the participated based on CD4 count was shown in Table 1. Between 1987 and 2016, data on 4402 patients who have CD4 cell count within three months after diagnosis, as well as no missing information, were included in this study. The mean age of study participants was 34.8 ± 10.4 years and 3030 (68.8%) were male. Most of the participants were married (48.5%) and jobless (39.5%). In addition, most participants were in baseline WHO clinical stage of I (45.4%), and mode of transmission in 47.8% patients was sexual contact.

The log-likelihood, AIC and RMSE for the Negative Binomial (NB) model were lower than two other models (Table 2). The estimate of r for NB and Generalized Poisson was 0.68 (S.E. 0.01) and 0.95 (S.E. 0.001) respectively which were significantly different from null value 0 (P < 0.001), therefore, NB and generalized Poisson models are favored over the Poisson. These results imply that the Negative Binomial model outperformed the Poisson and Generalized Poisson models for the used data set.

Output of NB model is presented in Table 3. Male gender was negatively associated with CD4 count. All the age group of the diagnosis has a significant effect. For all age groups, the CD4 counts decrease with increasing age group (P < 0.05). Marital status, Vocation status, and education level had not significant association with CD4 counts (P > 0.05). The transmission way of AIDS has a significant effect on CD4 counts. The patients infected by inject drug user, unsafe sex and blood transition had significantly lower CD4 counts than mother to child transition people. The patients with WHO clinical stage (II, III, IV and unknown) had significantly lower CD4 counts than the people with WHO clinical stage of I. Year of HIV diagnosis had a significant effect on the CD4 counts. The patients with year of HIV diagnosis before 2006 had significantly larger CD4 counts than people who their year of HIV diagnosis was after 2006. TB co-infection had a negative significant effect on CD4 counts (P < 0.05).

**Table 3:** Negative Binomial regression coefficients for factors affecting the initial number of CD4 cell counts

Variable	Coefficient	Standard Error	Z Score	P_value	95% Conf. Interval
Intercept	7.08	0.02	71.69	< 0.001	(6.88, 7.30)
Age					
< 30	Reference				
30–40	− 0.26	0.03	− 8.44	< 0.001	(− 0.32, − 0.20)
40–50	− 0.34	0.04	− 8.74	< 0.001	(− 0.42, − 0.27)
> 50	− 0.36	0.06	− 6.59	< 0.001	(− 0.47, − 0.26)
Sex					
Female	Reference				
Male	− 0.11	0.04	− 2.98	0.003	(− 0.18, − 0.04)
Marital status					
Single	Reference				
Married	0.01	0.03	0.28	0.78	(− 0.06, 0.07)
Divorced	0.02	0.04	0.54	0.587	(− 0.06, 0.11)
Window	− 0.18	0.11	− 1.71	0.086	(− 0.39, 0.03)
Unknown	− 0.18	0.11	− 1.71	0.086	(− 0.39, 0.03)
Education					
Illiterate	Reference				
Primary School	0.01	0.05	0.13	0.898	(− 0.10, 0.11)
Secondary School	0.05	0.05	0.84	0.404	(− 0.06, 0.15)
High School	0.04	0.06	0.71	0.476	(− 0.07, 0.15)
Academic	− 0.01	0.07	− 0.12	0.90	(− 0.15, 0.13)
Unknown	− 0.06	0.06	− 0.95	0.34	(− 0.17, 0.06)
Job					
Employed	Reference				
Unemployed	− 0.01	0.03	− 0.28	0.78	(− 0.07, − 0.28)
Unknown	− 0.02	0.03	− 0.57	0.569	(− 0.08, 0.05)
Transmission					
Mother to child	Reference				
Inject Drug User	− 0.45	0.09	− 5.21	< 0.001	(− 0.62, − 0.28)
Unsafe Sex	− 0.43	0.09	− 4.95	< 0.001	(− 0.59, − 0.26)
Blood Transfusion and Unknown	− 0.57	0.09	− 6.27	< 0.001	(− 0.75,− 0.39)
Year of HIV diagnosis					
Before 2006	Reference				
2006–2011	− 0.42	0.04	− 9.50	< 0.001	(− 0.51, − 0.34)
After 2011	− 0.39	0.04	− 8.67	< 0.001	(− 0.47, − 0.30)
WHO Clinical Stage					
Stage 1	Reference				
Stage 2	− 0.24	0.04	− 6.01	< 0.001	(− 0.31, − 0.16)
Stage 3	− 0.84	0.04	− 20.03	< 0.001	(− 0.93, − 0.76)
Stage 4	− 0.98	0.05	− 20.32	< 0.001	(− 1.08, − 0.89)
Unknown	− 0.19	0.04	− 5.23	< 0.001	(− 0.26, − 0.12)
TB co-infection					
No	Reference				
Yes	− 0.12	0.06	− 2.03	0.042	(− 0.01, − 0.23)
r	0.68	0.01			(0.66, 0.71)

Likelihood-ratio test of r = 0: chibar2 (01) = 7.5e + 05 Prob ≥chibar2 = 0.000

## Discussion

This study aimed to explore factors affecting baseline CD4 counts using Poisson, Negative Binomial and GPR regressions in HIV patients. In this study, we found that the Negative Binomial regression yielded the best fit. In addition, we found that CD4 counts decrease with increasing age group. The results of Tang et al. in [33], indicate that older patients are more likely to be diagnosed late based on CD4 counts. One of the reasons for late diagnosis in the elderly can be attributed to low education and information, as well as the low-risk perception of this disease in these people. Although it should be noted that the observed associations between CD4 cell count and subject characteristics at the time of HIV diagnosis will not be far from confounding factors such as the age of the PLHIV: patients who have been infected for a long time and get their diagnosis relatively late will have a relatively low CD4 cell count level but will also tend to be older.

The result of this study showed male gender was negatively associated with CD4 count. This finding approved by Mair et al. (2007) in Senegalese patients infected with other infections [10].

In the year 2016, Bruneau et al. used multiple quintile regression methods to identify the factors affecting the number of CD4 cells in France. The results of this study showed that higher age, male gender, external migrant patients, co-infection with hepatitis B and C viruses, rural residency and homosexual transmission method have a negative association with CD4 cell count. However, the statistical significance of these factors varied in different quantiles of CD4 [23].

Factors that affect the CD4 cell count in persons living with HIV (PLHIV) may indeed inform health care interventions or policies. However, establishing associations between CD4 cell count and risk factors based on cohort data will not directly lead to designing new health care interventions. Identifying these people and providing proper and adequate education in order to use prevention methods, change high-risk behavior or leave it can be an effective factor in preventing HIV infection in this group and ultimately its prevalence in society.

## Conclusions

One of the main priorities of public health in the field of HIV is to reduce the number of people who are treated with a lower CD4 cells counts at the time of diagnosis as a marker of HIV progression. The results of the present study confirm that certain groups of people such as older, men, people who inject drugs, people with unsafe sex and TB co-infection had lower initial CD4 counts. Therefore, awareness of such high-risk subsets for late detection can help guide policies and strategies to increase awareness and encourage people to early seek HIV testing and care.

## Limitation

We used registered data, some key variables had not been measured, and therefore we couldn’t either assess their effects on CD4 cell counts and or control their confounding effects. Many changes, such as demographic changes in cohort participants, changes in treatment policy, quality of care, HIV testing policies and promotions, as well as general public awareness and stigma towards PLHIV, may have emerged during the long time period of study. All these factors may be confounded with collected subject characteristics as well as the CD4 cell counts observed at HIV diagnosis. Moreover, all regression models used in this study are based on the mean response, while other regression such as quantile regressions can provide a more comprehensive analysis of the factors associated with CD4 cell counting.

## Data Availability

The datasets analyzed during the current study available from the corresponding author on reasonable request.

## Abbreviations

HIV: Human immunodeficiency viruses
AIDS: Acquired immunodeficiency syndrome
NB: Negative Binomial
PLHIV: Persons living with HIV
GPR: Generalized Poisson regression
